# Reply: Fetal safety of dydrogesterone: clarifying the role of pharmacovigilance

**DOI:** 10.1093/hropen/hoaf031

**Published:** 2025-05-21

**Authors:** Laurent Chouchana, Alexandra Henry, Mathilde Bourdon, Chloé Maignien, Charles Chapron, Jean-Marc Treluyer, Jean Guibourdenche, Pietro Santulli

**Affiliations:** Pharmacology Department, Regional Center of Pharmacovigilance, Cochin Hospital, AP-HP.Centre—Université Paris Cité, Paris, France; «Pharmacologie et évaluation des thérapeutiques chez l’enfant et la femme enceinte», Université Paris Cité, INSERM UMR-S1343, Paris, France; Pharmacology Department, Regional Center of Pharmacovigilance, Cochin Hospital, AP-HP.Centre—Université Paris Cité, Paris, France; «Pharmacologie et évaluation des thérapeutiques chez l’enfant et la femme enceinte», Université Paris Cité, INSERM UMR-S1343, Paris, France; Faculté de Santé, Université Paris Cité, Paris, France; Department of Gynaecology Obstetrics II and Reproductive Medicine, Cochin Hospital, AP-HP.Centre—Université Paris Cité, Paris, France; Department 3I « Infection, Immunité et inflammation », Institut Cochin, INSERM U1016, Paris, France; Faculté de Santé, Université Paris Cité, Paris, France; Department of Gynaecology Obstetrics II and Reproductive Medicine, Cochin Hospital, AP-HP.Centre—Université Paris Cité, Paris, France; Faculté de Santé, Université Paris Cité, Paris, France; Department of Gynaecology Obstetrics II and Reproductive Medicine, Cochin Hospital, AP-HP.Centre—Université Paris Cité, Paris, France; Department 3I « Infection, Immunité et inflammation », Institut Cochin, INSERM U1016, Paris, France; Pharmacology Department, Regional Center of Pharmacovigilance, Cochin Hospital, AP-HP.Centre—Université Paris Cité, Paris, France; «Pharmacologie et évaluation des thérapeutiques chez l’enfant et la femme enceinte», Université Paris Cité, INSERM UMR-S1343, Paris, France; Faculté de Santé, Université Paris Cité, Paris, France; Department of Hormonology, Cochin Hospital, AP-HP.Centre—Université Paris Cité, Paris, France; Faculté de Santé, Université Paris Cité, Paris, France; Department of Gynaecology Obstetrics II and Reproductive Medicine, Cochin Hospital, AP-HP.Centre—Université Paris Cité, Paris, France; Department 3I « Infection, Immunité et inflammation », Institut Cochin, INSERM U1016, Paris, France

Dear Editor,

We read with great interest the Letter to the Editor from Pabuccu *et al.* (2025) regarding our study on dydrogesterone and birth defect reporting (Henry *et al.*, 2025). Pabuccu *et al.*’s (2025) letter addresses important issues regarding drug safety and pharmacovigilance. We thank the Editor for giving us the opportunity to clarify key points and prevent any misunderstandings about our findings.

Our study, based on the World Health Organization (WHO) global safety database (VigiBase), identifies a disproportionate rate of reporting for birth defects, especially heart defects and hypospadias, with dydrogesterone use (Henry *et al.*, 2025). Disproportionality in birth defect reporting was consistent across several analyses, highlighting a potential safety signal which requires further assessment. In their letter, Pabuccu *et al.* (2025) stated that our findings do not reflect their clinical experience with dydrogesterone. In particular, they cite a recently published meta-analysis from Katalinic *et al.* (2024) as definitive evidence of dydrogesterone’s safety regarding birth defects. While we acknowledge that meta-analysis of randomized clinical trials (RCTs) is the highest level of evidence in medicine, we strongly disagree that this meta-analysis provides reassuring evidence. Instead, our study, based on disproportionality analyses, provides a foundation for further assessment of the fetal safety of dydrogesterone.

The primary role of pharmacovigilance is to monitor drug safety in real-world settings, complementing pre-marketing studies and playing a vital role in protecting public health (Croteau *et al.*, 2022). Among pharmacovigilance-related activities, safety signal detection and its assessment are crucial to allow further risk characterization and risk management. Clinical trials, even large RCTs, primarily designed to assess efficacy with safety as secondary endpoints, are inherently limited in their ability to detect rare or long-term adverse effects. As such, they lack the statistical power to detect rare events, such as birth defects, especially when the drug is used in a specific subpopulation (e.g. women undergoing ART). Regarding the aforementioned meta-analysis (Katalinic *et al.*, 2024), it includes data on 2690 livebirths: 1168 from cohort studies and 1512 from RCTs, in which the assessment of birth defects was only a secondary outcome, with no standardized criteria for classification, coding, or timing of assessment. Several limitations might be raised regarding the statistical and clinical relevance of the findings of this meta-analysis. First, the assessment of birth defect cases is a difficult task that requires advanced expertise. Standardized assessment is crucial to ensure accurate identification and classification (EUROCAT, 2003). Second, for some birth defects such as hypospadias, routine clinical assessment at birth may fail to detect the anomaly. In these cases, follow-up of the child during the first months of life is necessary to ascertain case identification. Third, owing to the incidence of hypospadias being ∼18 cases per 10 000 births (Bergman *et al.*, 2015) and considering the statistical distribution of rare events (Berlin *et al.*, 2008), detecting a risk such as a doubling of the baseline incidence rate (with reasonable confidence such as 80% power and a two-sided 0.05 significance level) would require around 50 000 exposed individuals. This vastly exceeds the number of live-births reported in the meta-analysis (Katalinic *et al.*, 2024). Foremost and finally, reporting of adverse events in clinical trials has been shown overall to be of poor quality or subjected to misinterpretation, and is sometimes associated with bias or selective reporting (Golder and Loke, 2008; Hughes *et al.*, 2014). In summary, RCTs, even if including pregnant women, are designed and performed to assess drug efficacy and are largely unpowered and inadequate to identify potential risks of birth defects.

As mentioned by a recent Letter to the Editor (Quadros *et al.*, 2024) in response to the publication of the meta-analysis (Katalinic *et al.*, 2024), a wealth of pharmacovigilance data, in addition to being a ‘sign giver’, may also serve as a cornerstone for identifying some serious adverse effects: for instance, birth defects. Indeed, history has shown that pharmacovigilance plays a crucial role in identifying drug-related teratogenic risks. In our study (Henry *et al.*, 2025), we used disproportionality analysis, also known as case–non-case analysis. This approach is widely used for signal detection, including within the context of pregnancy-related outcomes (Contejean *et al.*, 2023; Gougis *et al.*, 2023). Although it is designed as a case–control study, it is specifically intended for a pharmacovigilance purpose and its interpretation differs accordingly. Non-cases differ from controls in as much as they are constituted from individual case safety reports not having the adverse event of interest (in this instance, birth defects). The establishment of contingency tables assesses a potential statistical association between the drug exposure of interest and the reporting of the adverse event of interest, expressed by the reporting odds-ratio (ROR) and its 95% CI. However, it is important to note that the ROR should not be interpreted as the odds ratio (OR) in case–control studies: it does not quantify the magnitude of risk but rather signals a potential safety concern that warrants further assessment (Montastruc *et al.*, 2011; Fusaroli *et al.*, 2024). In other words, disproportionality in reporting may reflect a causal relationship between the drug and the adverse event of interest, although it is not definitive proof. Numerous pharmacovigilance studies have demonstrated the contribution of RORs in detecting valid safety signals (Piccinni *et al.*, 2011; Lepelley *et al.*, 2020; Touafchia *et al.*, 2021; Procacci *et al.*, 2025). In our study (Henry *et al.*, 2025), progesterone was used as an active comparator to reduce confounding by indication. Additionally, our sensitivity analyses, restricted to healthcare-professional reports and specific time periods, confirmed the consistency of the signal for disproportionate reporting.

Another common misunderstanding in pharmacovigilance is the belief that spontaneous reporting databases can be used to determine the actual incidence of adverse drug reactions. This is not possible: the main reasons being the under-reporting, which is inherently associated with spontaneous reporting, and the absence of knowledge regarding the number of patients treated (García-Abeijon *et al.*, 2023). Therefore, as opposed to what Pabuccu *et al.* (2025) suggest in their Letter, the number of reported birth defect cases relative to the total number of safety reports cannot be meaningfully compared to the general incidence rate of birth defects in the population. Furthermore, as mentioned by Pabuccu *et al.* (2025), we acknowledge that most of the birth defect cases with dydrogesterone were reported during the 2011–2020 period. However, this trend is not specific to dydrogesterone and is also observed with other ART-related drugs. More generally, rather than a time bias, this reveals the exponential increase of spontaneous reporting in the recent decade, demonstrating the growth in structured pharmacovigilance systems in several countries and a growing interest in pharmacovigilance.

An important point mentioned in the European Good Pharmacovigilance Practices for safety signal assessment is the biological or pharmacological plausibility (EMA, 2024). For instance, dydrogesterone has a potent progestogenic activity and high selectivity for progesterone receptors, and shows negligible or no agonistic activity at androgen receptors (Griesinger *et al.*, 2019). This contrasts with progesterone which has relatively high agonistic activity at androgen receptors. Fetal androgens are crucial, especially during the first trimester of pregnancy, for male sex differentiation (Kalfa *et al.*, 2009). Besides, it has been shown that hypospadias have unclear etiologies and might be caused by exogenous endocrine-disrupting chemicals with antiandrogenic effects in association with genetic determinants (EUROCAT, 2003). Hence, dydrogesterone may interfere with the complex hormonal regulation of male sex differentiation, potentially contributing to or increasing the risk of hypospadias.

Finally, we emphasize that our study does not claim to establish causality but rather highlights a potential safety signal that warrants further investigation (Henry *et al.*, 2025). Rather than dismissing pharmacovigilance findings, we advocate for a balanced interpretation that integrates pharmacovigilance data with epidemiological and experimental research. The history of drug safety has repeatedly shown that early pharmacovigilance signals, when appropriately investigated, lead to crucial regulatory and clinical decisions that protect patients. While dydrogesterone may offer benefits in ART, its widespread use in certain countries without well-defined clinical benefits should be reconsidered, and alternative therapeutic strategies should be explored. We call for large-scale, prospective cohort studies to ensure that the benefits–risks profile of dydrogesterone use during pregnancy is fully elucidated and evidence-based.
